# Reproduction of the Crystaline Lens

**Published:** 1867-10

**Authors:** 


### Reproduction of the Crystaline Lens.

From a pretty full *resume* of an important paper, read to the Academy of Sciences on 28th January, 1867, by M. Milliot, we may extract a few conclusions. The experiments were very numerous, extending over two years, and were performed on sheep, dogs, cats, rabbits, guinea-pigs, rats, and frogs. Extraction was generally performed by the flap operation, aided by free division of the anterior capsule.

He has proved the fact incontestably, that in animals a regeneration or re-production of the crystaline lens does frequently occur, even after complete removal. That this commences about the end of the second week after the operation, but is not completed till from five to twelve months, or even longer in aged animals. That the new lens is never so large as the old one. That sometimes the new one shows all the microscopic characters of the original lens; that more frequently it is an amorphous hyaline mass, which contains a small number of nuclei analogous to those of the cells known as the humor of Morgagni, sometimes fibrous tissue in laminæ, containing proliferating nuclei. Occasionally the cavity of the capsule becomes filled with vitreous humor, or, in cases where the iris has inflamed, with plastic lymph. That in human lenses affected by cataract, M. Milliot believes that except possibly in young subjects (which he has had no opportunity of verifying by dissection,) there is no reproduction of the lens. This is due partly to the age of the patients, partly to the changes which have been shown to occur in cataract, in the powers of endosmose and exosmose of the capsule, and through it on the nutrition of the crystaline lens. He has still doubts on this subject, and thinks it possible that in those cases where, in young subjects, vision is good without a cataract glass, that there really may be a reproduction of the lens in whole or in part.—*Ed. Med. Journal*, May, 1867.

[Drs. Cocteau and Leroy D'Etiolle over forty years ago (see Philadelphia Journal Med. and Phys. Sciences, 1827, vol. xiv, p. 384) instituted a number of experiments on rabbits to determine whether the lens, after removal, was reproduced, and they assert with an affirmative result. Dr. Barkhausen, of Berlin, however, repeated their experiments (see *Lancet*, 1831, p. 615) with a different result.]—*Ed. Am. Journ. Med. Sciences.*

